# Spent Medium Inhibits rVSV Infection

**DOI:** 10.3390/v18050557

**Published:** 2026-05-13

**Authors:** Rebecca Habisch, Johannes Georg Wieland, Sophia Kessler, Peter Neubauer, Jorge Soza-Ried, Eva Puschmann

**Affiliations:** 1 Boehringer Ingelheim, Viral Therapeutics Center, Untere Wiesen 22, D-88416 Ochsenhausen, Germany; 2 Chair of Bioprocess Engineering, Institute of Biotechnology, Technische Universität Berlin, Ackerstr. 76, ACK24, D-13355 Berlin, Germany

**Keywords:** rVSV, virus replication kinetics, infection, cell culture, cell density effect, attachment and entry

## Abstract

The cell density effect, defined as reduced cell-specific productivity above a critical cell density, remains a major limitation in virus manufacturing processes. While medium exchange prior to infection has been reported to mitigate this effect, the role of spent medium during the early phase of infection is poorly understood. Here, we show that spent medium conditioned by high-density HEK293 cultures inhibits infection with recombinant vesicular stomatitis virus (rVSV), even when infection is performed at low cell density. The strength of inhibition increased with the density and conditioning time of the donor culture and resulted in slower replication kinetics, thereby delaying the optimal harvest time and potentially reducing overall yield. Notably, the inhibitory effect was reversible when the virus was added to cells maintained in fresh medium, indicating that inhibition is mediated by the medium rather than intrinsic changes in the cells. We excluded pH effects within 7.1–8.0, nutrient depletion, and lactate/ammonium accumulation as primary causes. Removal of cell debris and extracellular vesicles by filtration (down to 0.02 µm) and size-based retention down to 3 kDa did not restore infection, and AUC indicated no major differences in particle distributions between fresh and conditioned media. Together, our data suggest an unidentified <3 kDa inhibitor in spent medium that partially suppresses rVSV infection and slows replication kinetics.

## 1. Introduction

High cell density cultivation is commonly pursued in virus manufacturing as it offers the potential for a higher titer yield and more efficient use of facility capacity. This approach could be crucial for the production of therapeutic viruses, for which qualified production sites are limited, and the cost of goods is a significant factor from a commercial perspective. However, the potential of cultivating high cell density is frequently limited by the cell density effect, a recurring phenomenon in which the cell-specific yield declines once cultures exceed a critical cell density [1,2,3]. In virus production, this creates several practical obstacles: increasing cell density does not necessarily translate into higher viral titers; process intensification can become unpredictable; and key operational decisions—most importantly, when to infect and when to harvest—may shift or become harder to define. Mechanistically, understanding the drivers of the cell density effect is therefore important not only for maximizing yields but also for improving the robustness and transferability of virus production processes. The extent and criticality of the cell density effect are specific to the culture medium used [2]. Zeng [3] described that in antibody production, it may be associated with nutrient availability. For virus production processes, multiple studies report that exchanging the medium prior to infection can mitigate the cell density effect or increase the critical cell density in recombinant vesicular stomatitis virus (rVSV) production [4,5]. Similarly, Dill et al. [6] showed in a study with foot-and-mouth disease virus that a 100% medium exchange can mitigate the decrease in absolute viral titers caused by higher cell densities. While this observation implies nutrient depletion as one of the main root causes, an effect due to the accumulation of metabolic and waste byproducts cannot be ruled out, as they might create an environment disadvantageous to virus infection and replication. Along the same lines, Pérez-Rubio et al. [7] showed that, in transfection-based virus production, transfection is inhibited by spent medium, but can be restored by removing extracellular vesicles. Although the benefits of perfusion on process yield have been recently demonstrated [5,8,9], to our knowledge no study has yet examined the impact of spent medium on early virus infection and replication kinetics. Here, we demonstrate that spent medium inhibits infection with rVSV, resulting in slower overall replication kinetics. This, in turn, delays the optimal harvest time and results in a lower production yield. We examined the role of extracellular vesicles on rVSV infection and found that exclusion of molecules or structures larger than 3 kDa does not influence the observed effect. We further exclude accumulation of lactate or ammonium, nutrient depletion, and pH as potential causes, narrowing down the search for inhibitory factors to compounds smaller than 3 kDa.

## 2. Materials and Methods

### 2.1. Cell Culture

HEK293 cells (Thermo Fisher Scientific, Waltham, MA, USA) were inoculated at 5 × 10^5^ cells mL^−1^, cultured in BalanCD (Fuji Film, Santa Ana, CA, USA), and passaged twice per week. The 60 mL cultures were incubated in 250 mL shake flasks (Corning, Corning, NY, USA) at 37 °C, 120 rpm with 5% CO_2_.

### 2.2. Conditioning of Spent Medium

Cultures were inoculated at 5 × 10^5^ cells mL^−1^ and cultured as described in Section 2.1. Spent medium was harvested after conditioning for the indicated period via centrifugation (180× *g*, 5 min, RT) and collection of supernatant. For simplicity, throughout the presented study, the medium spent is always described alongside the culture age, following inoculation at 5 × 10^5^ cells mL^−1^ (e.g., 6 day (6 d)-culture medium for spent medium conditioned for 6 days). For 2 d, 4 d, and 6 d cultures, average total cell densities were 2.1 × 10^6^ cells mL^−1^, 5.5 × 10^6^ cells mL^−1^, and 8.0 × 10^6^ cells mL^−1^, respectively.

### 2.3. Influence of Spent Medium on rVSV Replication Kinetics

To investigate the effect of spent medium on virus replication, HEK293 cells were either inoculated at 5 × 10^5^ cells mL^−1^ in spent medium, fresh medium, or Dulbecco’s phosphate-buffered saline (PBS). Cells were immediately infected with either recombinant vesicular stomatitis virus pseudotyped with the glycoprotein of lymphocytic choriomeningitis virus (rVSV) at MOI 0.1, or with rVSV carrying Green Fluorescent Protein (GFP) as the reporter gene (rVSV-GFP) at MOI 1. Only infected cells express the viral cargo GFP and can thus be visualized by fluorescent microscopy. Kinetics experiments were performed in 125 mL shake flasks (Corning) with a working volume of 30 mL under the same conditions as described in Section 2.1.

### 2.4. GFP Imaging

200 µL of cell suspension was transferred from an infected culture to a well on a 96-well plate. Plates were incubated in a BioSpa (Agilent, Santa Clara, CA, USA) at 37 °C with 5% CO_2_ and imaged using the Cytation 5 plate reader (Agilent). Images were processed using the FIJI distribution of ImageJ (v 1.54) [10]. Quantification of fluorescence intensity was performed using ImageJ. A representative area was selected for calculating the background intensity, which was then subtracted from the average intensity of each image.

### 2.5. Infectious Particles

For analysis of infectious titer, 0.5 mL samples were collected and treated for virus release as described by Gautam et al. [11]. Subsequently, samples were centrifuged at 1000× *g* for 5 min at RT, and the supernatant was recovered and stored at −80 °C until analysis. TCID_50_ was performed according to Hochdorfer et al. [12] and as described previously [13]. 

### 2.6. Sampling for Genomic Titer

Following the sample treatment for virus release [11], samples were centrifuged at 180× *g* for 5 min. The cell pellet was washed in PBS once, resuspended in PBS to the original volume, and frozen at −80 °C until further processing. RNA extraction and qPCR were performed as described previously [13]. 

## 3. Results

**Figure 1:**
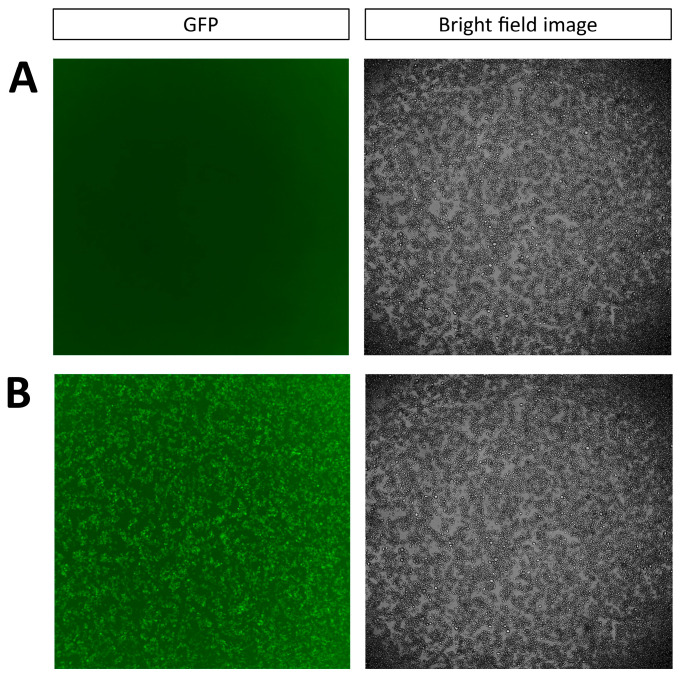
Investigation of GFP expression after infection with rVSV-GFP at MOI 1. (**A**) HEK293 cells originating from a low-cell-density culture in 6 d culture medium. (**B**) HEK293 cells originating from a high-cell-density culture in fresh medium.

Cells and media from high-cell-density cultures were separated and used independently in a series of experiments to investigate their influence on virus productivity. To evaluate the effect of media, rVSV was spiked to cells originating from a low-cell-density culture (below 3 × 10^6^ cells mL^−1^), and infection was carried out in a medium previously conditioned by a 6 d cell culture. To explore the effect of high-cell-density-cultured cells, rVSV was spiked to cells from high-cell-density cultures, and the infection was carried out in fresh medium for infection and the duration of the experiment. GFP expression, which reflects virus replication, was then monitored via imaging at 24 h following infection. Upon inoculation and infection in spent medium, cells originating from a low-cell-density culture did not express GFP (fluorescence intensity = 0.01 a.u., Figure 1A). Cells taken from high-cell-density cultures inoculated and infected in fresh medium produced observable amounts of GFP (fluorescence intensity = 6.60 a.u., Figure 1B).

**Figure 2:**
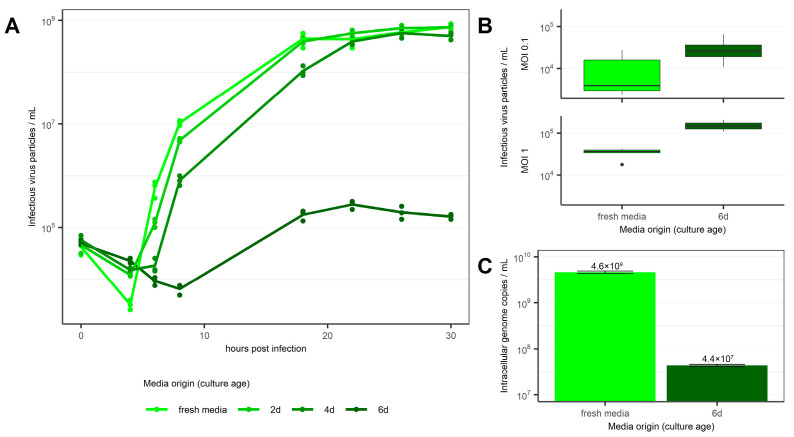
rVSV titers in HEK293 cells infected in fresh and spent media. (**A**) rVSV infectious titer (TCID_50_ mL^−1^) after infection at MOI 0.1 (*n* = 3 parallel cultures). (**B**) rVSV titers at 4 hpi with MOIs 0.1 and 1 in fresh or 6 d culture medium (*n* = 7 independent experiments). (**C**) Intracellular genomic copies at 4 hpi after infection with MOI 1 (*n* = 2 technical replicates).

Based on this, rVSV replication kinetics were evaluated in cells infected and cultured in spent medium collected at different cell culture confluences (i.e., from 2 d, 4 d, and 6 d cell cultures). Results were compared to the replication kinetics observed when the virus infected cells in fresh medium (Figure 2), which reached a maximum titer of 7.54 × 10^8^ TCID_50_ mL^−1^ after 30 h. Cultures inoculated with spent medium from 2 d cultures achieved comparable total titers, but at initially slower kinetics (Figure 2A). Cultures infected in spent medium conditioned for 4 days reached slightly lower maximum titers of 5.71 × 10^8^ TCID_50_ mL^−1^ and exhibited even slower kinetics. At 4 h post-infection (hpi), it was possible to observe a delay and a less intense virus uptake by all cells infected in spent medium (Figure 2A). This observation was further confirmed when 6 d spent media was used for infection (Figure 2A), even when different MOIs were used (Figure 2B).

**Figure 3:**
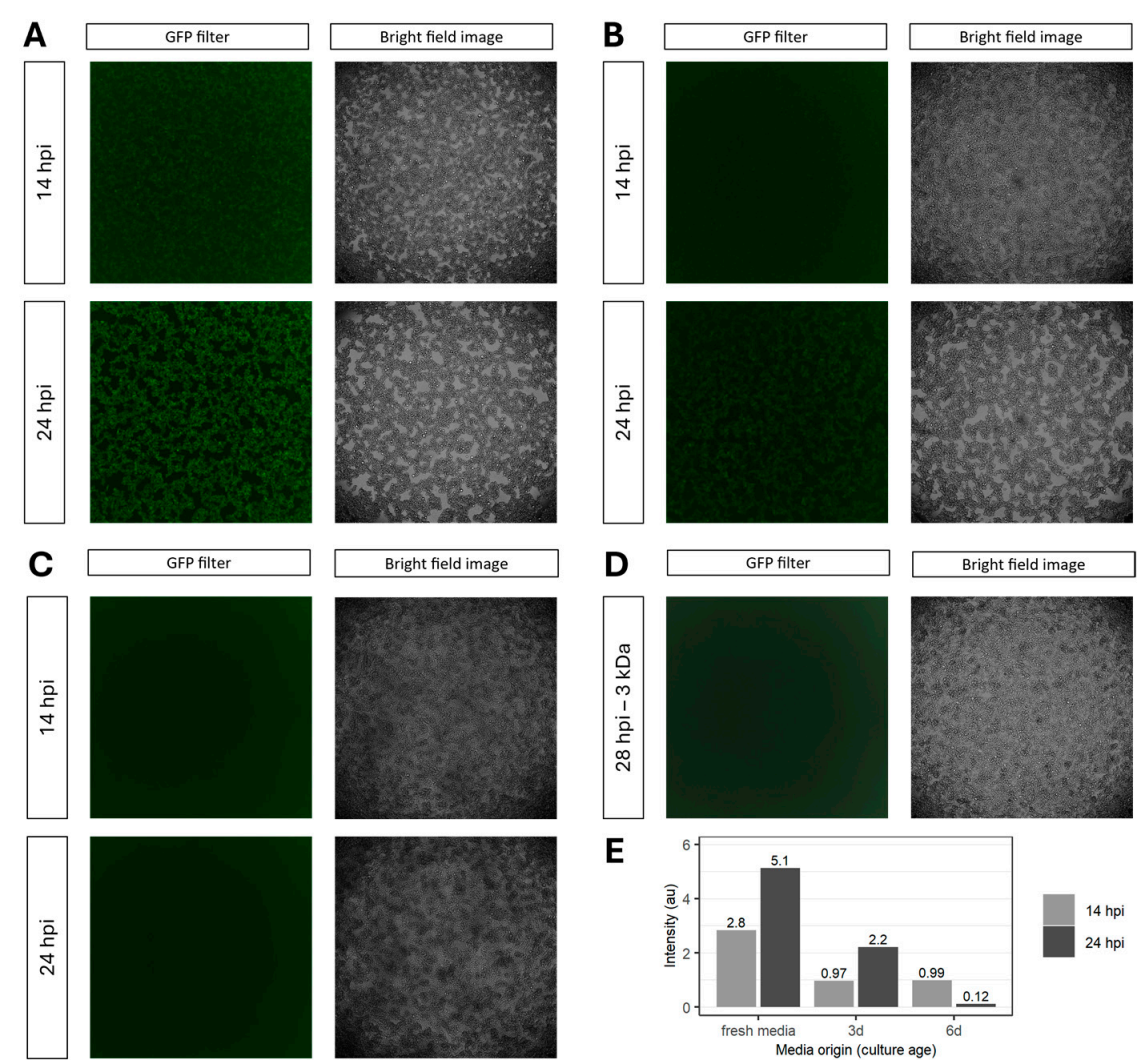
Investigation of GFP expression in HEK293 cells infected with rVSV-GFP at MOI 1. Image with GFP filter and as a brightfield image after infection in (**A**) fresh medium (*n* = 8 independent experiments). (**B**) The cell culture supernatant of a 3-day culture (*n* = 2 independent experiments). (**C**) The cell culture supernatant of a 6-day culture (*n* = 7 independent experiments). (**D**) The permeate of a 3 kDa filtration of C (*n* = 1 independent experiment). (**E**) Calculated fluorescence intensity of (**A**–**C**).

To investigate which step of virus replication is influenced by the spent medium, we quantified intracellular genome copies after infection. At 4 hpi, usage of 6 d culture media resulted in 4.38 × 10^7^ intracellular genome copies mL^−1^. This represents a significant titer reduction (*p* < 0.01) of up to two orders of magnitude compared to infections carried out in fresh medium (Figure 2C). As the TCID_50_ assay measures infectious particles in the supernatant, while any particles associated with the cell are previously removed via centrifugation, these results suggest inhibition of either cell attachment or cell entry and, consequently, an impaired infection in spent medium. Next, we confirmed this observation by inspecting GFP expression after infection using rVSV-GFP in fresh, 3 d and 6 d culture media (Figure 3). At 14 hpi, GFP was only detectable in cells infected in fresh medium. GFP intensity increased until the final time point at 24 hpi (Figure 3A,E). At this time point, GFP expression was also detectable in cells infected in 3 d culture medium (Figure 3B,E). Conversely, no GFP expression was observed at either time point when 6 d culture medium was used (Figure 3C,E). Consistent with the measurements of intracellular genomic copies (Figure 2B), these results confirm a correlation between the extent of media consumption and/or the increase in byproducts in extracellular medium and the efficiency of virus infection. To exclude the influence of extracellular vesicles and inhibitory proteins as the cause of the observed inhibition of infection, spent media were filtered (range of pore size between 3 kDa and 200 µM) before being used in infections. Filtration of the 6 d culture medium through a 3 kDa pore size filter prior to infection did not rescue the GFP expression (Figure 3D). We subsequently analyzed the macromolecular composition of unfiltered 6 d culture and fresh medium using analytical ultracentrifugation (AUC) (Supplementary Figure S1). The sedimentation coefficient distribution was determined by centrifuging both samples at 42,000 rpm and measuring the radial extinction at 280 nm in 5 min intervals. The results showed comparable sedimentation coefficient distribution for both samples. This means there is a similarity regarding mass, shape, and density distribution of particles absorbing at 280 nm (i.e., proteins) in the solution of respective samples.

**Figure 4:**
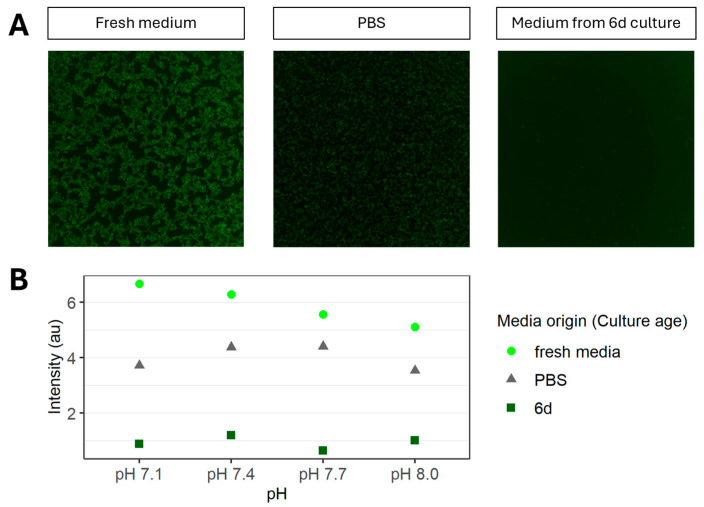
Investigation of GFP expression in HEK293 cells infected with rVSV-GFP in different media with different pH levels. (**A**) Images with GFP filter. (**B**) Calculated fluorescence intensity (*n* = 1).

We further evaluated whether pH differences or nutrient depletion might account for the inhibitory effect observed with the 6 d culture medium upon infection. The pH in the 6 d culture medium ranged from 7.1 to 8.0. After adjusting fresh medium to various pH values within this range, infection with rVSV-GFP still led to observable GFP expression (Figure 4 and Supplementary Figure S2). Analysis of lactate and ammonium levels showed no correlation with the decrease in the final titer or in replication kinetics. Finally, inoculation and infection in PBS resulted in observable GFP expression (Figure 4 and Supplementary Figure S2).

## 4. Discussion

Our findings demonstrate that spent medium inhibits rVSV infection of HEK293 cells. This observation confirms and extends previous reports in adenovirus and foot-and-mouth disease virus (FMDV) production that have shown a negative impact of cell densities on viral yield even when different media compositions were used [6,14]. While typical batch culture processes for commercial manufacture of viruses use optimal cell densities in the range of 1 to 5 × 10^6^ cells mL^−1^ at infection, as demonstrated recently [15,16], Shen et al. demonstrated that virus productivity decreased when HEK293 cultures were infected at a density above 3 or 4 × 10^6^ cells mL^−1^ [14]. To further explore the underlying mechanism of this effect, we focused on the early stage of the infection process and observed reduced replication kinetics when infection was performed in the presence of spent media from high-cell-density cultures. Interestingly, cells originating from the same high-density culture can be infected efficiently when infection is performed in fresh medium. Indeed, the extent of infection inhibition increases if the medium used for infection comes from older (i.e., 6 d) cultures. We have observed that infection is not completely abolished (Figure 2C), and it can be restored when the same viruses are used to infect cells cultured in fresh medium. The data presented in Figure 2A,B is based exclusively on viral particles in the media, while cell-associated or internalized viral particles are removed from the suspension via centrifugation prior to analysis. Thus, the difference that can be observed in viral titers at 4 hpi between fresh and spent media suggests an inhibiting effect during viral attachment or during internalization.

It has been described that lower pH (pH < 6.0) induces an irreversible conformational change in the glycoprotein spike complex of the lymphocytic choriomeningitis virus (LCMV), resulting in a loss of infectivity [17]. In the current study, the pH in the spent media ranged between 7.1 and 8.0. An adjustment of PBS or fresh medium to this pH range had no effect on the detection and level of GFP from the infected cultures (Figure 4). These results rule out pH as a root cause of the limited yield observed in infections performed in the presence of spent media. Importantly, rVSV was able to infect cells kept in PBS, although to a lesser extent when compared to fresh medium conditions. Altogether, these observations suggest that accumulation of extracellular byproducts rather than the pH, or a lack of nutrients alone, seems to have a significant impact on the success of viral infection. We investigated whether the removal of exosomes, cell debris, and proteins larger than 3 kDa would restore the infection capability of rVSV-GFP in spent medium, as observed for transfection-based virus production [7]. Nevertheless, no GFP expression could be detected upon infection in the filtered permeate of spent medium, indicating no improvement regarding infection efficiency. We also confirmed via AUC that the sedimentation of molecular components present in fresh medium and 6 d culture medium shows no significant difference. This indicates similarity in terms of mass, shape, and density distribution of particles in solution (Supplementary Figure S1). It is therefore likely that the inhibition of infection is caused by the accumulation of an inhibitory compound smaller than 3 kDa. Dilution or removal of these factors could explain the subsequent boost in virus production seen after media exchange during repeated harvest, fed-batch, or perfusion studies [13]. The effect on replication kinetics and the varying times required to reach the maximum harvest titer suggest that future studies should include multiple harvest time points, rather than one fixed time point, when comparing viral titers across cultures with different cell densities at the time of infection. In summary, we have demonstrated that spent medium partially inhibits infection and thereby slows virus replication kinetics. The presented study suggests this infection inhibition is not caused by pH, proteins, or residuals larger than 3 kDa. More experiments are needed to investigate the influence of nutrient depletion and media components on viral infection. These findings offer valuable insights for future investigations aimed at optimizing production parameters in this field.

## Data Availability

The data presented in this study are available on request from the corresponding author.

## Supplementary Materials

The following supporting information can be downloaded at: https://www.mdpi.com/article/10.3390/v18050557/s1; Figure S1: Sedimentation coefficient distributions for fresh and 6 d culture medium at 42 000 rpm. Extinction was measured at 280 nm. Figure S2: Investigation of GFP expression in HEK293 cells infected with rVSV-GFP in different media with different pH.

## Abbreviations

The following abbreviations are used in this manuscript:

rVSV	Recombinant vesicular stomatitis virus
rVSV-GFP	rVSV carrying Green Fluorescent Protein (GFP) as reporter gene
