# Supplementary figures and images for: Spent Medium Inhibits rVSV Infection
Source: Viruses. 2026 May 13;18(5):557. doi: 10.3390/v18050557 (PMC13211489; doi:10.3390/v18050557)

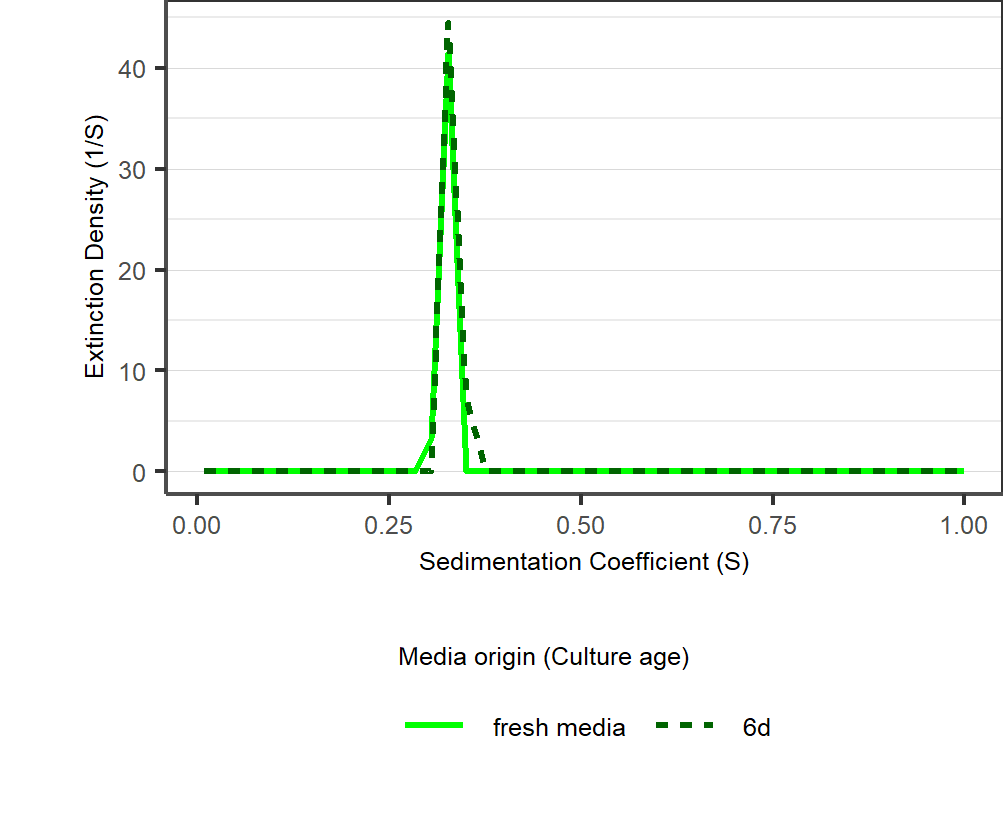

Supplement: Supplementary file 1 [file viruses-18-00557-s001.zip › Supplementary Figure S1.tif]
